# Supplementary material for: Plant Polyphenols Inhibit Functional Amyloid and Biofilm Formation in Pseudomonas Strains by Directing Monomers to Off-Pathway Oligomers
Source: Biomolecules. 2019 Oct 26;9(11):659. doi: 10.3390/biom9110659 (PMC6920965; doi:10.3390/biom9110659)
Supplement: Supplementary file 1 [file biomolecules-09-00659-s001.pdf]

Plant polyphenols inhibit functional amyloid and biofilm formation in *Pseudomonas* strains by directing monomers to off-pathway oligomers

Zahra Najarzadeh,
Hossein Mohammad-Beigi, Jannik Nedergaard Pedersen, Gunna Christiansen,
Thorbjørn Vincent Sønderby, Seyed Abbas Shojaosadati, Dina Morshedi,
Kristian Strømgaard, Georg Meisl, Jan Skov Pedersen and
Daniel E. Otzen

Supplementary Figures

Najarzadeh et al. Figure S1

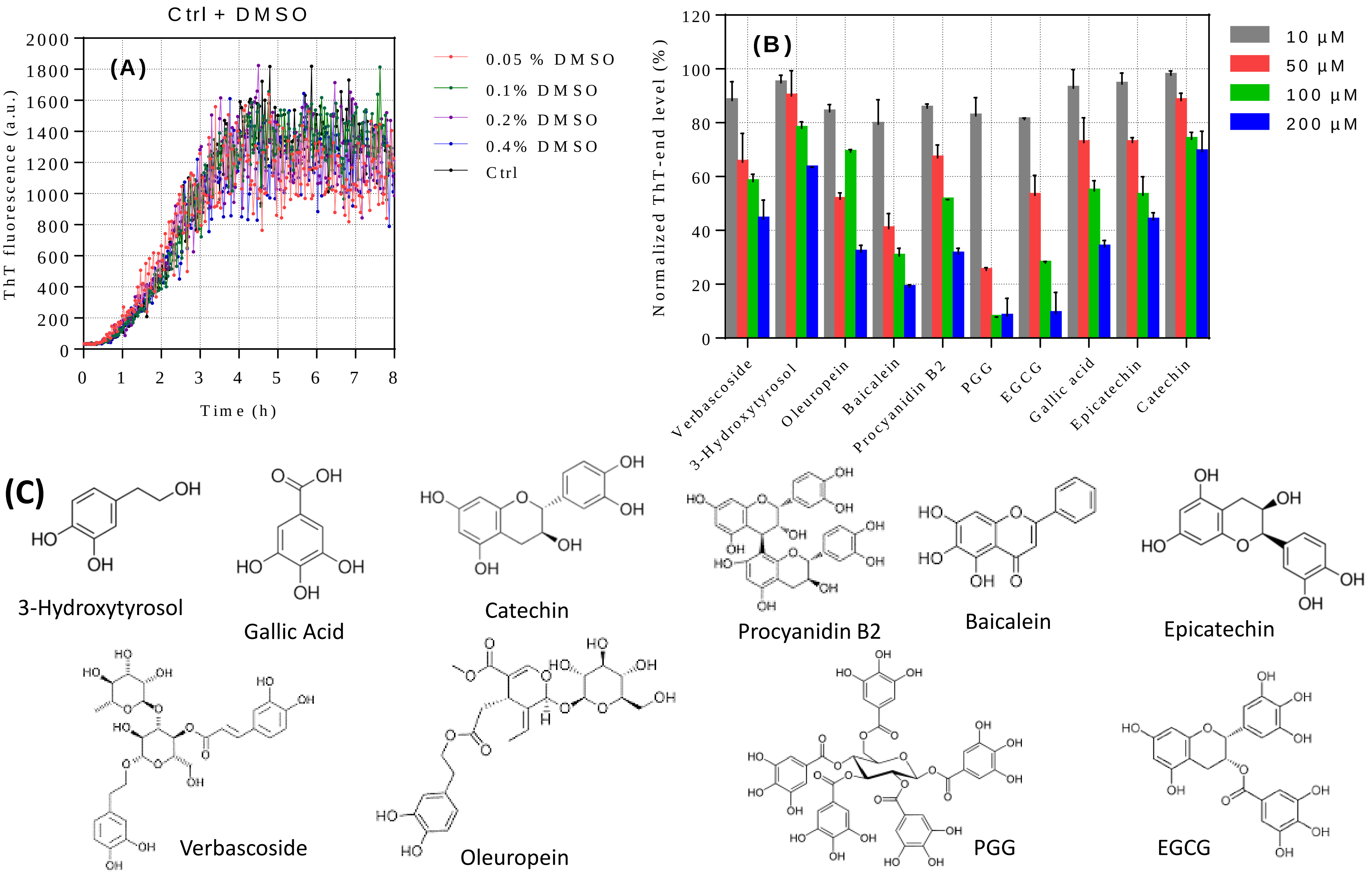

Figure S1. (A) Effect of DMSO on fibrillation of FapC (30 µM) , DMSO at concentrations that used in this study didn't show any significant effect. (B) Normalized end levels of ThT fluorescence after incubation of 30 µM FapC with 10-200 µM of different polyphenols for 24 h. Data are normalized to the absence of polyphenols. PGG, EGCG, baicalein, procyanidin B2 and oleuropein selected as the top best inhibitors for the next experiments. (C) Structures of compounds used in this study.

Najarzadeh et al. Figure S2

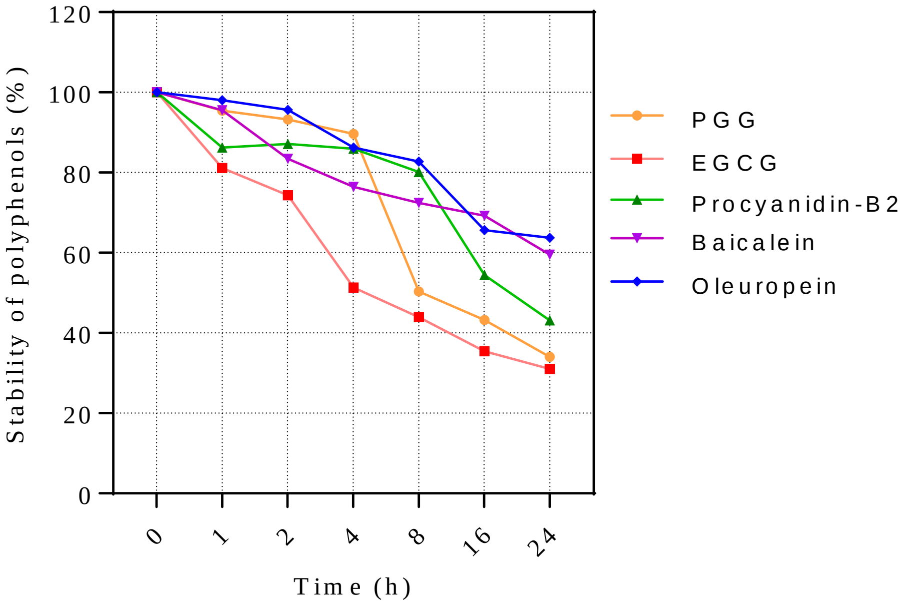

Figure S2. Reverse phase-HPLC (RP-HPLC) of polyphenols used to estimate their stability in 50 mM Tris pH 7.5 at 37 °C. Samples were loaded on a C18 column and the area under peak curves at different time points is shown.

Najarzadeh et al. Figure S3

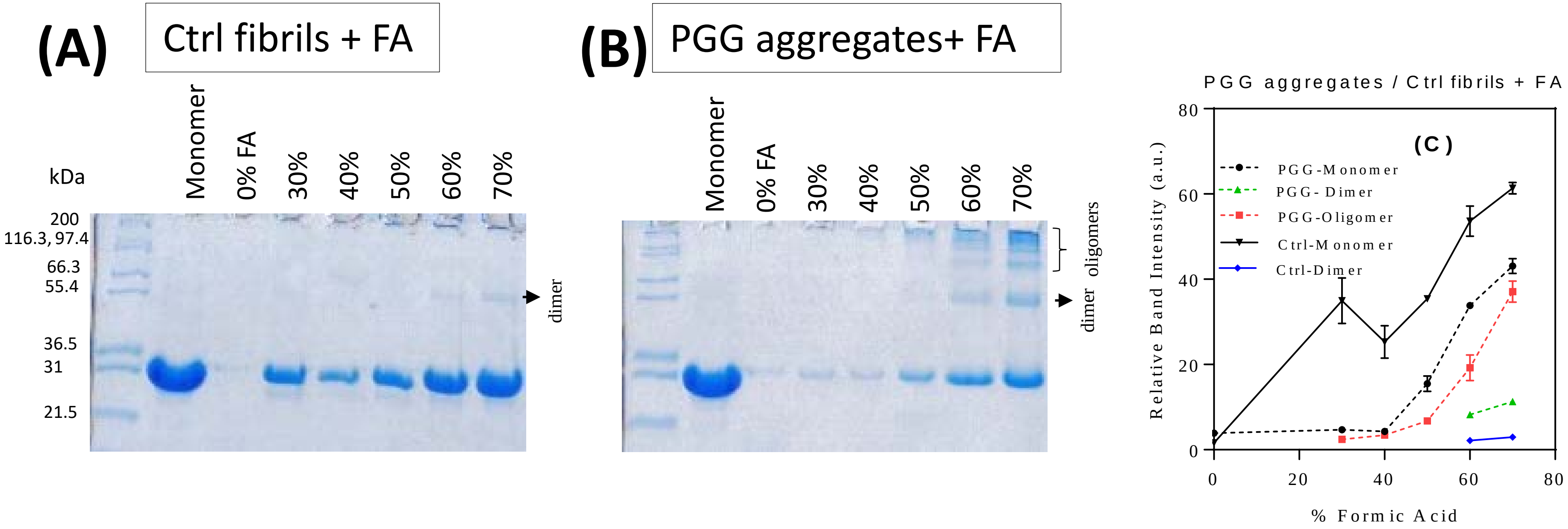

Figure S3. Assessment of the stability of FapC aggregates using formic acid. FapC aggregates made in (A) absence and (B) presence of 120 µM PGG were incubated in different concentrations of formic acid (FA). In the absence of FA, the aggregates do not dissociate and thus cannot enter the gel. (C) Quantification of bands from figures A and B with ImageJ software, dashed lines are from PGG-aggregates and solid lines are from the PGG-free control.

Najarzadeh et al. Figure S4

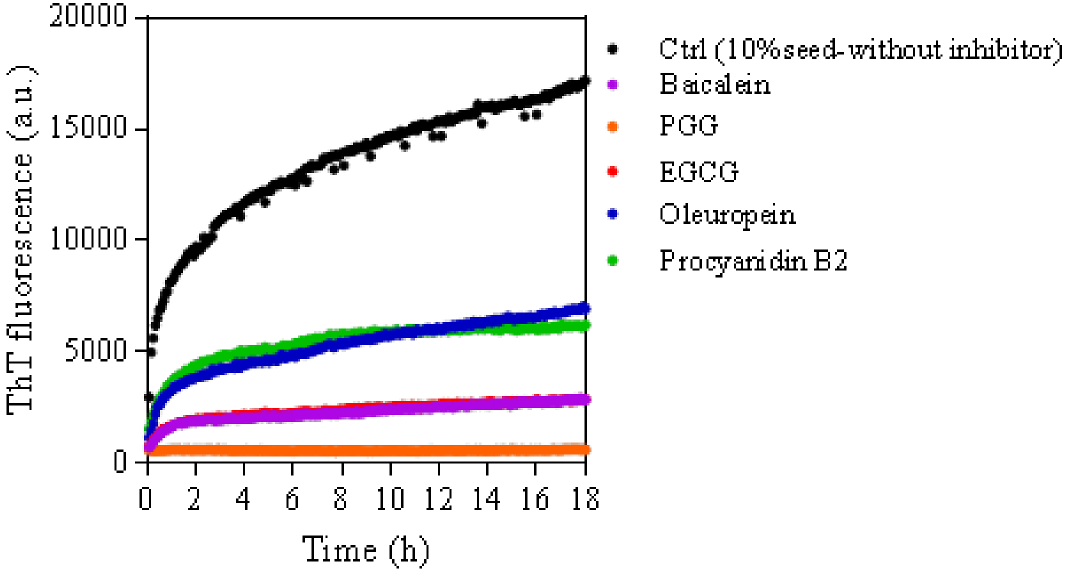

Figure S4. Seeding experiments (ThT endpoint data summarized in Fig 2A). Seeds: FapC fibrils. Solution: 30 µM monomeric FapC together with 120 µM polyphenols.

Najarzadeh et al. Figure S5

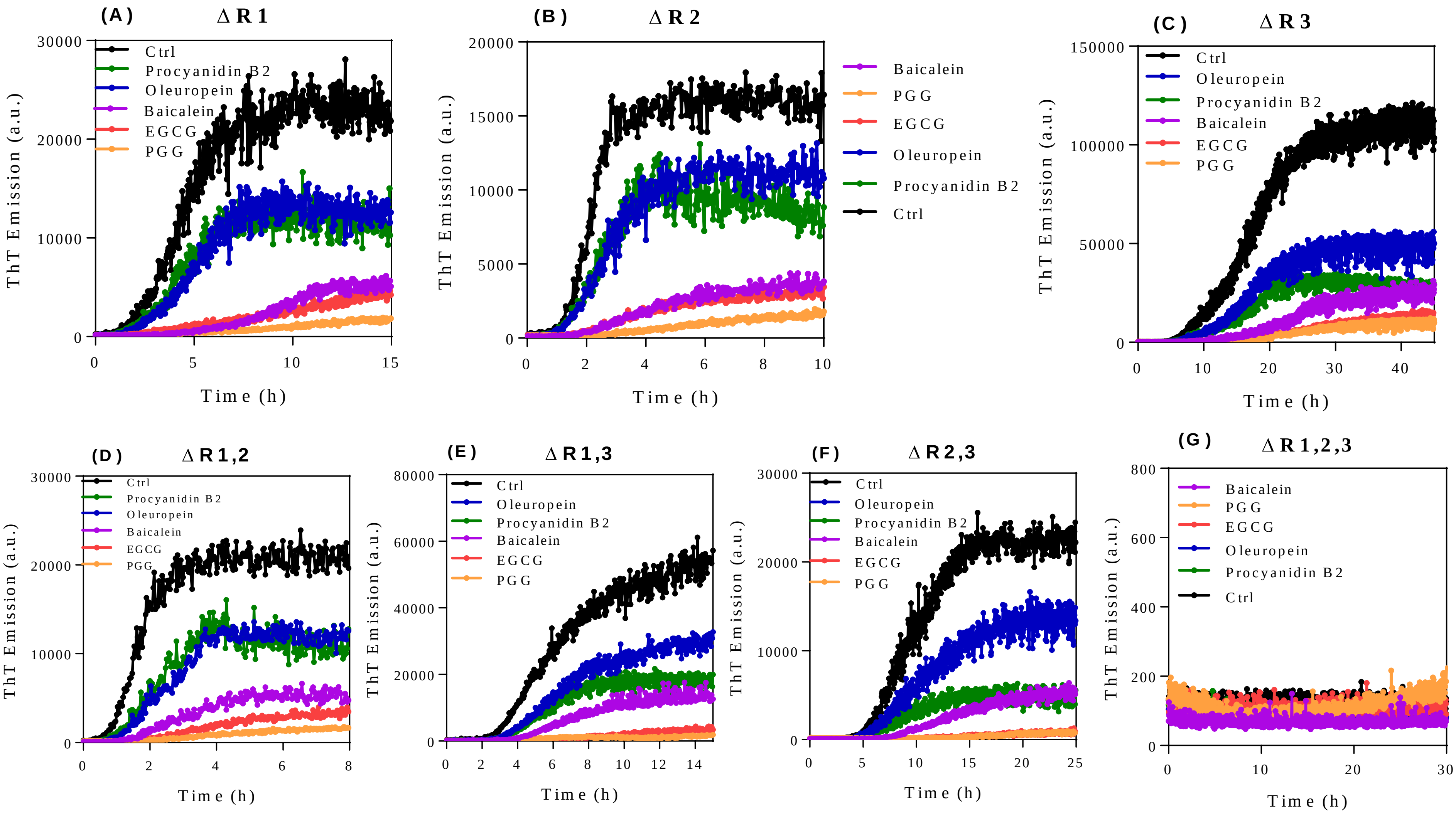

Figure S5. ThT-assay of different repeat-deletion mutants of FapC-UK4 incubated with polyphenols. The legends are the same for all graphs. For each mutant, the indicated amyloidogenic repeats are removed. Mutants lacking R3 showed significantly longer lag phases than the other repeats.

Najarzadeh et al. Figure S6

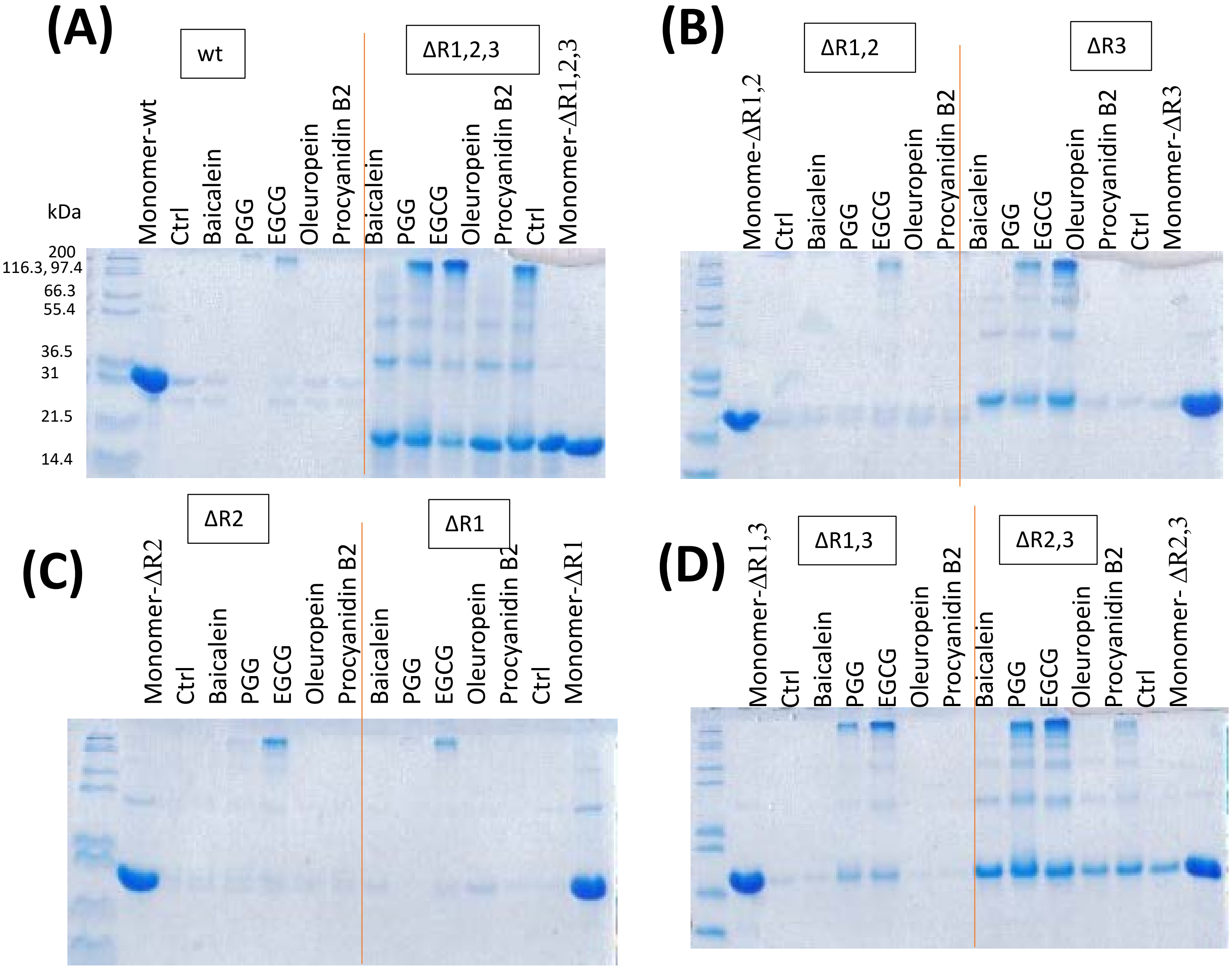

Figure S6. SDS-PAGE analysis of different mutants incubated with 120 μM polyphenols for 48 hrs. PGG and EGCG clearly induce formation of more oligomers than the other polyphenols, particularly for mutants ΔR1,2,3, ΔR3 and ΔR2,3.

Najarzadeh et al. Figure S7

(A)

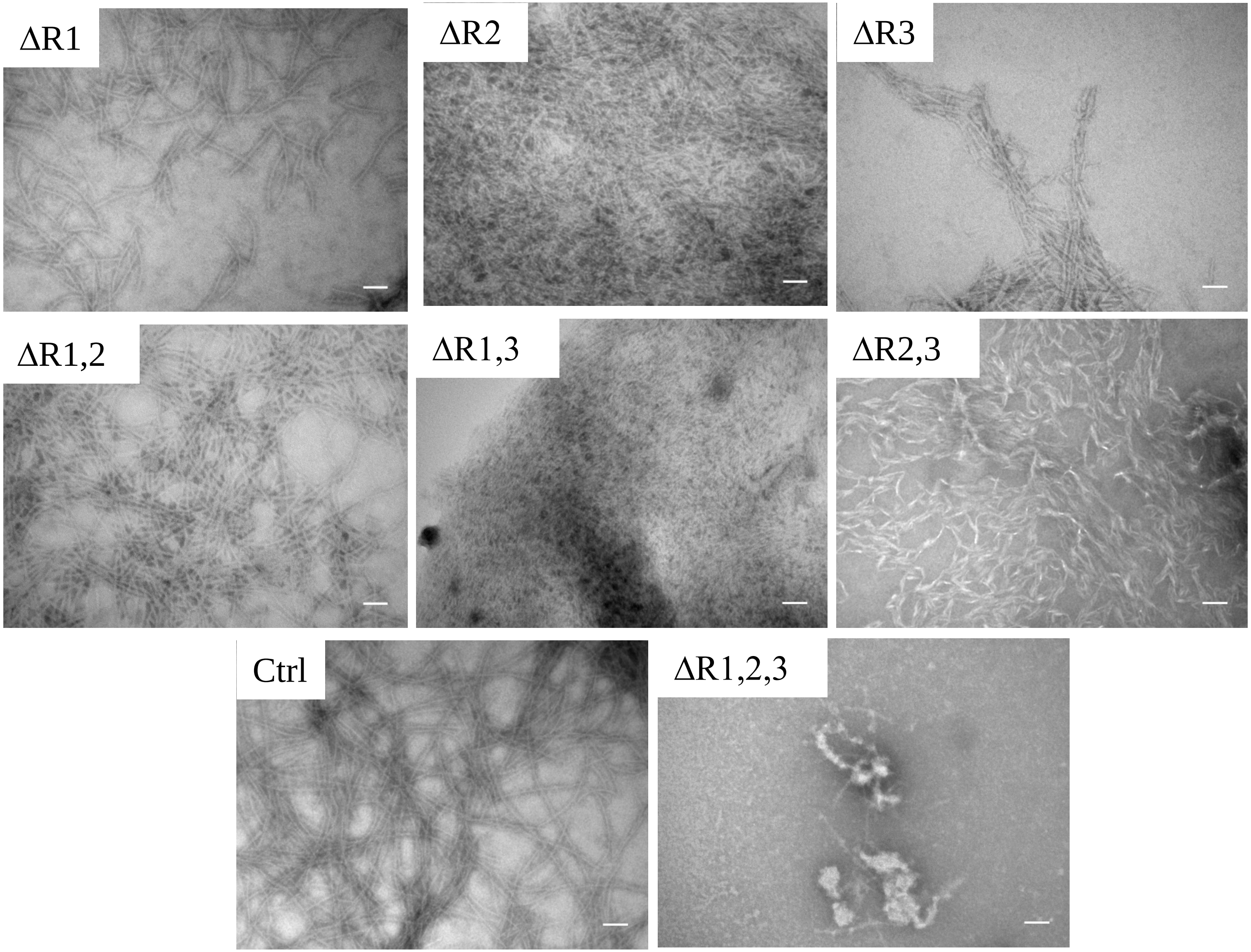

Figure S7. TEM images of FapC wild type (wt) and mutants incubated (A) without polyphenols. Scale bars are 100 nm.

Najarzadeh et al. Figure S7

(B)

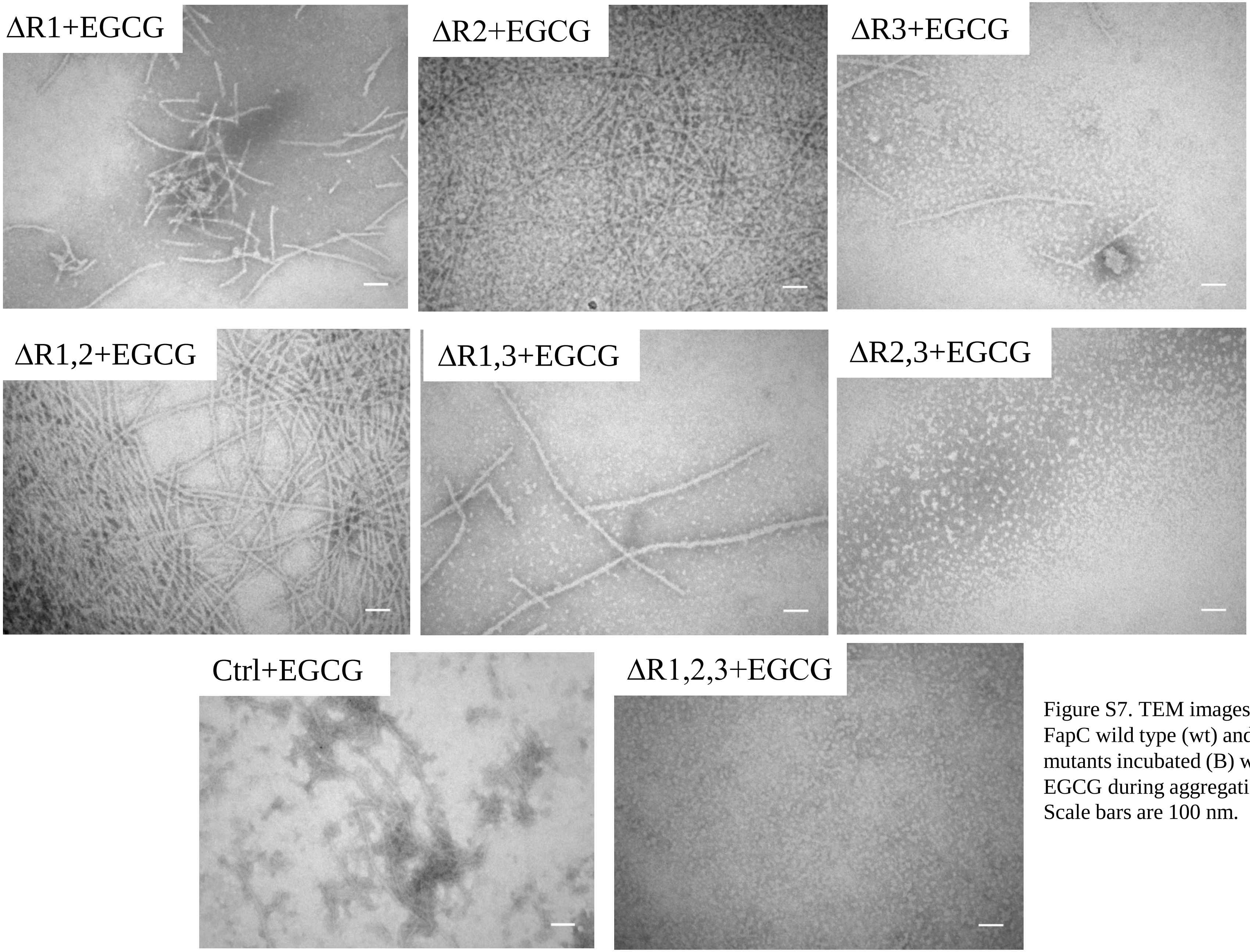

Figure S7. TEM images of FapC wild type (wt) and mutants incubated (B) with EGCG during aggregation. Scale bars are 100 nm.

Najarzadeh et al. Figure S8

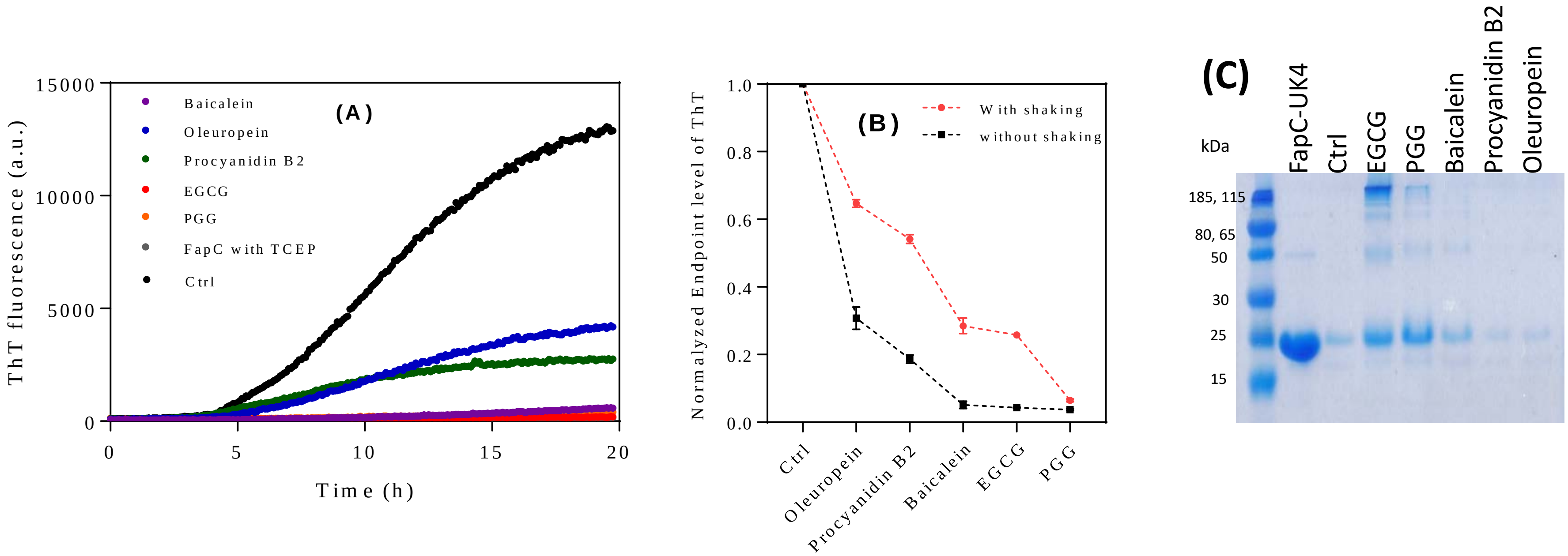

Figure S8. (A) ThT time profile for FapC-UK4 incubated without agitation in the absence or presence of 120 µM polyphenols. (B) Endpoint ThT levels for FapC-UK4 fibrillation with and without polyphenols and with and without shaking. (C) SDS-PAGE of the entire sample (including both soluble and insoluble species) after 20 hrs incubation.

Najarzadeh et al. Figure S9

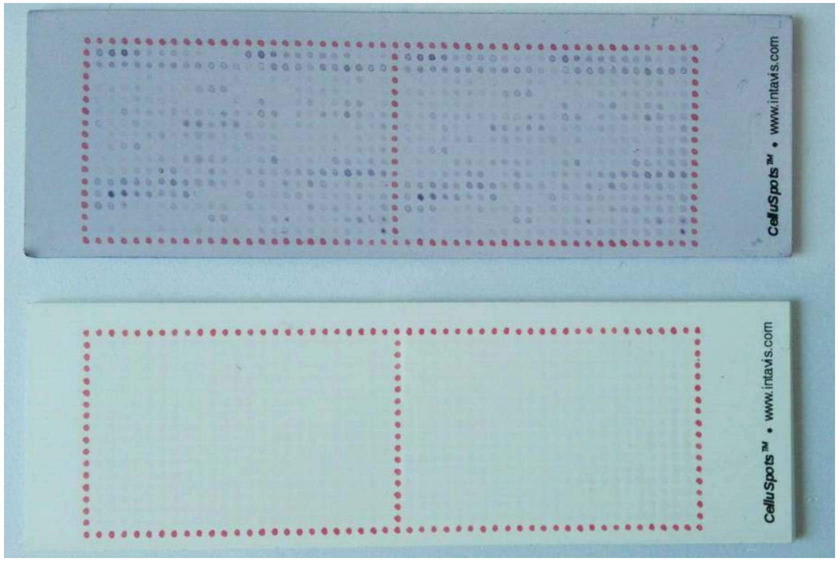

+ EGCG
+ NBT

- EGCG
+ NBT

Figure S9. Peptide array with and without EGCG. Both arrays have been stained with NBT, which reacts with the quinone group on EGCG and accordingly does not stain in the absence of EGCG (lower array).

Najarzadeh et al. Figure S10

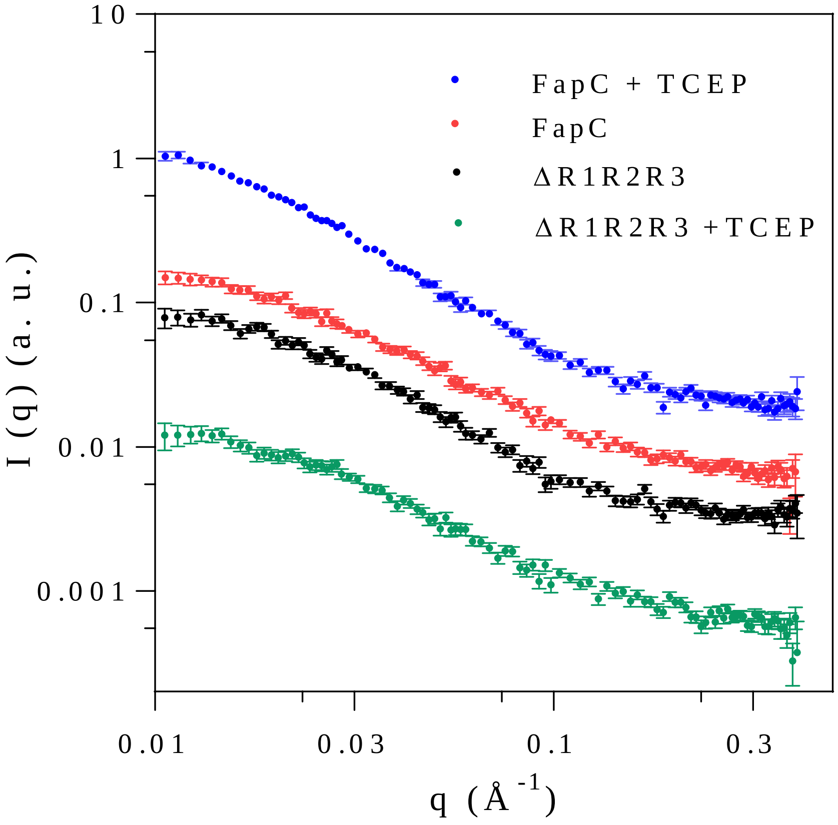

Figure S10. SAXS data of the freshly desalted samples of FapC, and the mutant ΔR1R2R3 both without and with TCEP
